# Complementary and Integrative Medicine for Neurocognitive Disorders and Caregiver Health

**DOI:** 10.1007/s11920-022-01355-y

**Published:** 2022-08-13

**Authors:** Sarah A. Nguyen, Hanadi Ajam Oughli, Helen Lavretsky

**Affiliations:** Department of Psychiatry and Biobehavioral Sciences, David Geffen School of Medicine, University of California, Los Angeles; 760 Westwood Plaza, Los Angeles, CA 90095 USA

**Keywords:** Cognition, Dementia, Alzheimer’s, Complementary, Alternative, Integrative medicine, Caregivers

## Abstract

***Purpose of Review*:**

Integrative medicine is the practice of combining conventional medical treatments with “alternative” or “complementary” therapies. Integrative psychiatry is a holistic, person-centered approach to neuropsychiatric disorders that emphasizes a person’s physical, emotional, interpersonal, behavioral, nutritional, environmental, and spiritual dimensions to achieve well-being. Older adults are more prone to physical injury, interpersonal loss, chronic illnesses, and physical and cognitive decline that can manifest as anxiety, depression, with functional decline and inability to care for self. Additionally, stress of caring for older adults with dementia can adversely affect caregivers’ health. Although integrative approaches are perceived as safer and less stigmatizing, it is important to understand the risks and benefits of such therapies for older adults with neurocognitive disorders and their caregivers.

***Recent Findings*:**

Here, we summarize the results of the recent clinical trials and meta-analyses that provide evidence for integrative approaches to treating older adults with cognitive disorders and their caregivers which include the use of diet and supplements, and mind–body therapies.

***Summary*:**

Dietary and mind-body therapies have become increasingly popular and show the strongest evidence of effectiveness for cognitive disorders and caregiver stress. Vitamins and supplements are the most popular integrative intervention, but there is mixed evidence supporting their use and the concern for herb (supplement)-drug interactions. While there is increasing popularity of integrative treatments, information to guide clinicians providing care for older adults remains limited, with variable scientific rigor of the available RCTs for a large number of commonly used integrative interventions particularly for cognitive disorders and caregiver stress and well-being.

## Introduction

The terms “alternative,” “complementary,” and “integrative” are often used interchangeably. However, the National Center for Complementary and Integrative Health (NCCIH) explicitly defines these terms: “Alternative” medicine refers to a set of practices of medicine (e.g., traditional, Oriental, mind–body, etc.) without sufficient evidence base that is used in place of conventional medicine; meanwhile, “complementary” medicine signifies non-mainstream practice that is used together with conventional medicine [1••]. “Integrative” medicine refers to the use of conventional and complementary approaches in a coordinated way to target specific disorders or underlying neurobiological processes.

**Fig. 1:**
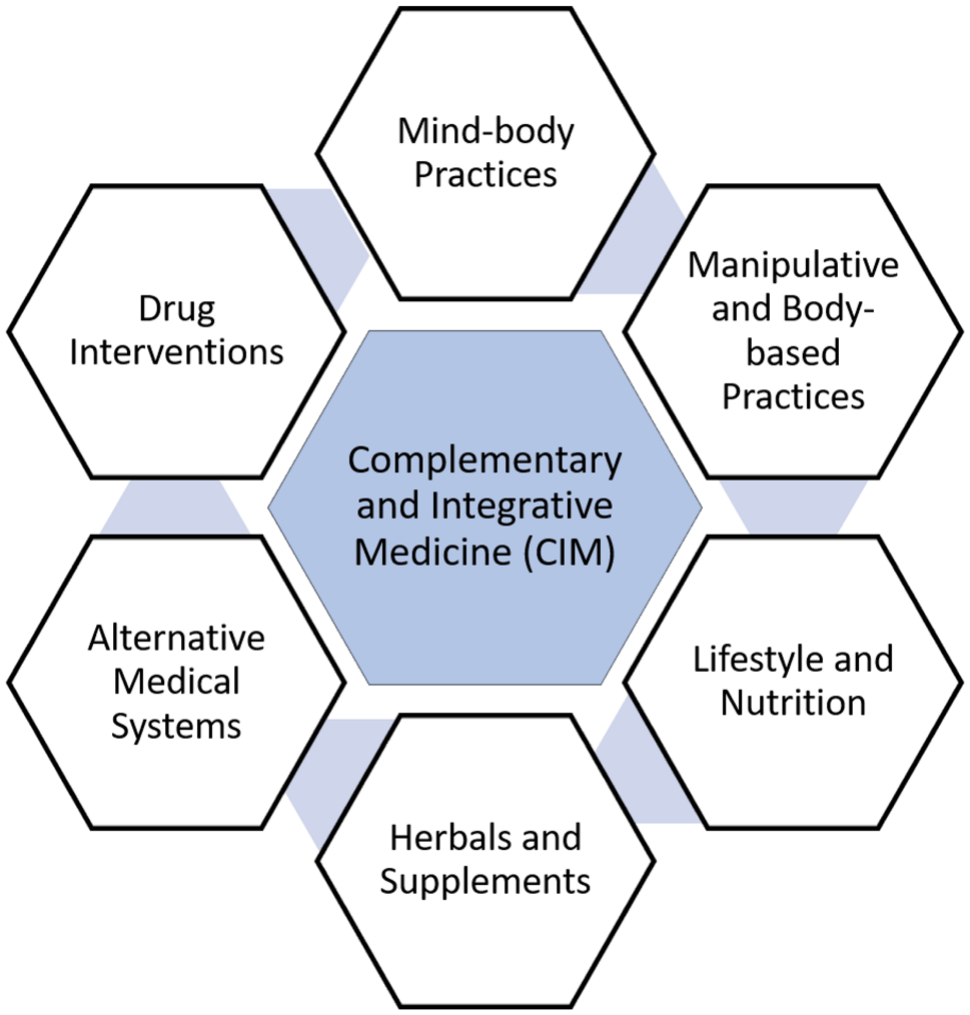
Types of complementary and integrative approaches

Acceptance and use of complementary and integrative medicine (CIM) therapies are growing since it is often perceived as a more natural and safer option compared to conventional medicine. Among the Baby Boomers cohort born in 1946–1964 and later, these interventions are rapidly gaining popularity, particularly in the USA [2, 3]. Integrative approaches incorporate the belief that well-being is a state of balance encompassing spiritual, physical, and mental/emotional functioning. Appropriate nutrition, exercise, sleep habits, and the ability to regulate stress responses help achieve this balanced, healthy lifestyle. Figure 1 presents the categories of CIM practices.

The NCCIH recognizes three categories for complementary and integrative approaches: (1) mind and body practices (massage therapy, meditation, yoga, acupuncture, chiropractic/osteopathic manipulation, hypnotherapy, Tai Chi, qi gong, healing touch, and relaxation exercises); (2) natural products (herbal medicines, botanicals, vitamins, minerals, probiotics, and other dietary supplements); (3) other complementary approaches (indigenous healing practices, Chinese medicine, Ayurvedic medicine, homeopathy, and naturopathy). This review will focus on the evidence supporting these practices used in managing neurocognitive disorders (NCDs) and for caregiver stress and well-being.

## Complementary and Integrative Medicine Use in Neurocognitive Disorders and for Caregiver Health

While CIM can be used to promote healthy aging, they are also applicable to the disorders of aging including chronic mental, cognitive, and physical conditions. The use of CIM offers less invasive, more cost-effective, scalable, and culturally acceptable therapies [4]. A joint survey conducted by the American Association of Retired People (AARP) and the NCCIH showed the use of CIM increasing rapidly and exceeding a prevalence of 53% among those aged 50 years and above [5].

The Neurocognitive Disorders (NCD) category in the Diagnostic and Statistical Manual of Mental Disorders, 5th Edition (DSM-5) encompasses a group of disorders in which the primary clinical deficit is in cognitive function regardless of the underlying etiology or age of onset. The term “dementia” is still commonly used to refer to Alzheimer’s disease, frontotemporal lobar degeneration, Lewy body disease, vascular dementia, and others. These subtypes are distinguished based on combination of time course, characteristic cognitive domains affected, and associated symptoms. We will use the terms, NCD and dementia, interchangeably unless a dementia subtype is specified.

Alzheimer’s disease, the most common subtype of dementia, affects more than 6 million Americans, with 1 in 3 older adults dying from Alzheimer’s or another dementia [6]. Additionally, these patients eventually require additional caregiving. Family caregivers provide physical and emotional assistance to individuals with dementia and often endure emotional, physical, and financial burden that leads to chronic stress and physical decline [7, 8••]. There are an estimated 11 million people in the USA providing unpaid care for those with dementia, the equivalent of nearly $257 billion in caregiving [6]. It is well-known that caregiver burden is associated with poor physical health, compromised immune function, social isolation, loneliness, chronic sleep disturbances, stress, anxiety, and depression [9]. Therefore, it is important to also focus on caregiver health and well-being as well as those who are being cared for. Complementary and integrative therapies are commonly used to reduce stress and improve sleep, physical and psychological health and well-being, with increasing interest utilizing these interventions for older adults [1, 10–12]. The studies of the impact of CIM interventions on cognition and caregiver stress and well-being are summarized in Table 1.

**Table 1:** Study characteristics of CIM Interventions

**Reference**	**Study design**	**Intervention**	**Study sample**	**Findings**
***Mind–body and body-based therapies***				
Chobe et al. [16]	Systematic review	Yoga for cognition	13 RCTs, *N* = 1115, adults ages 55–92	Yoga-based intervention have some positive evidence on attention, executive functions and memory among cognitive variables compared to active controls but not specific for individuals with MCI or dementia Average Delphi scores (indication of risk bias) of RCTs = 3.92, suggesting moderate study quality
Bhattacharyya et al. [17••]	Meta-analysis	Yoga for cognition and cognition with MCI	11 RCTs, *N* = 912 adults with cognitive impairment	Significant beneficial effects on memory (Cohen's *d* = 0.38), executive function (Cohen’s *d* = 0.40), and attention and processing speed (Cohen’s *d* = 0.33) with no adverse effects
Yang et al. [46]	Systematic Review and Meta-analysis	Tai chi on cognition in MCI	11 RCTs, *N* = 1061, mean age > 60 with MCI	Tai Chi can have moderate to significant benefits for global cognitive function (SMD = 0.35) in older adults with MCI, as suggested by previous studies. Regarding the specific domain of cognition, Tai Chi may improve to a small to medium or significant degree memory and learning (SMD = 0.37), mental speed and attention (SMD = 0.51), ideas, abstraction, figural creations, and mental flexibility (SMD = 0.29), and visuospatial perception (SMD = 0.29)
Hsieh et al. [19]	Randomized Control Trial	Virtual Reality Tai Chi on Cognition in MCI and dementia	*N* = 60; ages ≥ 65 years and MMSE 11–26	VRTC exercise program posed a significant protective effect on abstract thinking and judgment, aerobic endurance, lower extremity endurance, balance, and gait speed but only the ability of abstract thinking and judgment was maintained for cognitive function in the VRTC group after 6 months Average movement accuracy score of 3 months significantly predicted improvement in the total CASI score (*β* = 0.426, *t* = 2.432, *p* = 0.023) but no significance at 6 months
Marciniak et al. [20]	Randomized Controlled Trial	MBSR on Cognition in MCI	*N* = 28 individuals with MCI	MSBR group showed a significant decrease in GDS score between baseline and visit 2 (*p* = 0.03) and baseline and visit 3 (*p* = 0.0461). No significant differences were observed in cognitive tests or scores between baseline and visit 2 or visit 3
Wells et al. [21]	Randomized Controlled Trial	MBSR for MCI	*N* = 14 adults, ages 55–90 with MCI	No significant differences detected between MBSR and control in ADAS-cog change from baseline
Lenze et al. [23]	Randomized Controlled Trial	MBSR for cognitive dysfunction in anxiety	N = 34 adults, ages 65 years or older with significant anxiety-related distress plus subjective cognitive dysfunction	MBSR showed a trend toward improvement in all cognitive measures but there was no advantage for 12-week MBSR: Cohen’s *d* for worry reduction was 1.47 for 8-week and 0.48 for 12-week
Wetherell et al. [24]	Randomized Controlled Trial	MBSR on Cognition	*N* = 103 adults, ages 65 or older with anxiety, depression, or SND	Mindfulness group experienced greater improvement on a memory composite score (*p* = .046) but groups did not differ on change in cognitive control
Oken et al. [25]	Pilot randomized control trial	MBSR/MBCT on caregiver stress	*N* = 31 caregivers aged 45–85 years of close relatives with dementia	Significant effect found on self-rated caregiver stress with MBCT intervention
Whitebird et al. [26]	Randomized Control Trial	MBSR on caregiver stress and caregiver depression	*N* = 78 caregivers for individuals with dementia	MBSR was more effective at improving overall mental health, reducing stress, and decreasing depression MBSR participants showed immediate improvement and reported better mental health (*p* = .007), reported lower stress (*p* = .007) and depression (*p* = .005) and maintained improvement at 6-month follow up, but the difference between MBSR and control groups at 6 months did not differ significantly
Brown et al. [27]	Pilot randomized control trial	MBSR on caregiver stress and caregiver depression	*N* = 38 caregivers for individuals with dementia	MBSR participants reported significantly lower levels of perceived stress and mood disturbance at post-intervention relative to standard social support participants but did not differ at 3-month follow-up
Watson et al. [54••]	Randomized Controlled Trial	Aromatherapy on Agitation in Dementia	*N* = 49; 39 with dementia and 10 without dementia	Lemon Balm was more effective in reducing agitation (*p* = .04) and physical non-aggressive behavior (PNAB) (*p* = .02) in participants without dementia. Lavender was more effective in reducing PNAB (*p* = 0.04) in dementia
Ballard et al. [55]	Randomized Controlled Trial	Aromatherapy on Agitation in Severe Dementia	*N* = 72 with agitation in the context of severe dementia	60% of participants treated with lemon balm versus 14% of placebo treated group experienced a reduction in 30% of Cohen-Mansfield Agitation Inventory (CMAI), with an overall improvement in agitation of 35% in participants receiving lemon balm oil and 11% in those treated with placebo (Mann–Whitney U test; Z = 4.1, *p* < .0001)
***Aerobic exercise***				
de Oliveira Silva et al. [28]	Randomized Controlled Trial	Exercise on Cognition in MCI and dementia	*N* = 28 adults ages 65 and older with MCI or AD	Significant difference only in the simple task mobility test (ΔCG: − 0.18 ± 0.53; ΔEG: − 1.05 ± 0.57; *p* = 0.03) and in the verbal fluency (ΔCG: − 1.30 ± 2.49; ΔEG: 3.16 ± 1.72, *p* = 0.05) of the elderly with MCI, showing a beneficial effect of the multimodal exercise in this group (not AD group)
Song et al. [29]	Meta-analysis	Exercise on Cognition	11 RCTs; *N* = 929, adults ages 18 and older with MCI	Physical exercise had beneficial effects for global cognition in MCI (SMD) = 0.30, 95% confidence interval (CI): 0.10–0.49, *p* = 0.002]. Further subgroup analysis demonstrated that aerobic exercise program are consistently associated with medium effect size (SMD: 0.54–0.58)
Huang et al. [30••]	Meta-analysis	Exercise on Cognition	71 trials; *N* = 5606 participants	All types of exercise were effective in increasing or maintaining global cognition, and resistance exercise had the highest probability of being the most effective intervention in slowing the decrease in global cognition: (standard mean difference (SMD) = 1.05, 95% confidence interval (95%CI): 0.56–1.54), executive function (SMD = 0.85, 95%CI: 0.21–1.49), and memory function (SMD = 0.32, 95%CI: 0.01–0.63) in patients with cognitive dysfunction Only resistance exercise showed significant effects on memory function for patients with MCI (SMD = 0.35, 95%CI: 0.01–0.69)
Lamb et al. [31]	Randomized Controlled Trial	Moderate to high intensity exercise training for individuals with mild-moderate dementia	*N* = 494 participants diagnosed with dementia	A moderate to high intensity aerobic and strength exercise training program does not slow cognitive impairment in people with mild to moderate dementia
Yan et al. [32••]	Meta-analysis	Sedentary Lifestyle on Dementia Risk	18 cohort studies; *N* = 250,063 adults and *N* = 2269 adults with dementia	Sedentary behavior was significantly associated with increased risk of dementia (*RR* = 1.30; 95% CI: 1.12–1.51)
***Vitamins and natural supplements***				
Burckhardt et al. [39]	Cochrane Review	Omega-3 polyunsaturated fatty acids	3 RCTs; 632 participants with mild to moderate AD	No evidence of a benefit from omega-3 PUFAs on cognitive function when measured at 6 months with Alzheimer’s Disease Assessment Scale–Cognitive subscale (SMD − 0.02, 95% CI − 0.19 to 0.15; 566 participants, 3 studies) or MMSE (MD 0.18, 95% CI − 1.05 to 1.41; 202 participants; 2 studies)
Levkovitz et al. [44]	Randomized Controlled Trial	SAMe on MDD	N = 46 with MDD administered adjunctive oral SAMe	There was a greater improvement in the ability to recall information (*p* = 0.04) and a trend toward statistical significance for greater improvement in word-finding (*p* = 0.09) for patients who received adjunctive SAMe than placebo
Yang et al. [46]	Meta-analysis	Ginkgo biloba in cognitive impairment (MCI and AD)	2608 participants in 21 RCTs, adults	Gingko biloba in combination with conventional medicine was superior in improving MMSE scores at 24 weeks for patients with AD (MD 2.39, 95% CI 1.28 to 3.50, *p* < 0.0001) and mild cognitive impairment (MD 1.90, 95% CI 1.41 to 2.39, *p* < 0.00001), and ADL scores at 24 weeks for AD (MD -3.72, 95% CI − 5.68 to − 1.76, *p* = 0.0002)
Malouf et al. [37]	Cochrane Review	Vitamin B 6 on cognition	109 health older adults in 2 RCTs	No statistically significant differences between treatment with vitamin B6 supplementation versus placebo was found on cognition or mood

*MCI* mild cognitive impairment, *SMD* standardized mean difference, *VRTC* virtual reality Tai Chi, *MMSE* Mini-Mental Status Exam, *CASI* Cognitive Abilities Screening Instrument, *MBSR* Mindfulness-based stress reduction, *GDS* geriatric depression scale, *ADAS*-*cog* Alzheimer’s Disease Assessment Scale, cognitive subscale; *SND* subjective neurocognitive difficulties, *MBCT* mindfulness-based cognitive therapy, *AD* Alzheimer’s disease, *PUFA* polyunsaturated fatty acids, *CI* confidence interval, *SAMe S*-Adenosyl-l-methionine, *MD* mean difference, *ADL* activity of daily living

## Mind–Body Practices

Mind–body therapies (MBT) can be divided into mindful exercise and meditative practices. Mindful movement exercises include yoga, tai chi, and qigong. Meditative practices include progressive relaxation, mindfulness, meditation, and acceptance therapies. According to a 2017 National Health Survey, there is rising popularity with 5–10% increased use of yoga, meditation, and chiropractic care, with growing research evidence supporting the use of MBTs as minimally invasive, cost-effective approaches for management of stress and neurocognitive disorders [13••]. In contrast to pharmacological approaches, MBTs aim to teach patients life-long skills for stress-reduction that may continue to confer benefits through self-regulation, which is also particularly helpful for caregiver stress and well-being.

Yogic practices consist of different components, i.e., yogic postures (asanas), breathing practices (pranayama), and meditation (Dharana and dhyana) have had some positive evidence on attention, executive functions, and memory among cognitive variables [14, 15]. A 2020 systemic review found 13 randomized controlled trials examining the impact of yoga on cognition and mental health [16]. These RCTs examined different domains of cognition, including executive function, memory, attention, and language, as well as depression, anxiety, stress, and mood and found some positive evidence in improving attention, executive functions, and memory of cognition. However, this review did not specifically look at adults with MCI or dementia when considering effects on specific cognitive domains. These studies were also limited by small number, with no limitations placed on the type, duration, and frequency of the intervention. A more rigorous meta-analysis examined yoga can serve as a preventative mind–body therapy for management of cognitive decline in older adults [17••]. Twelve studies, including 912 participants, were included, with eleven being RCTs. Results revealed significant beneficial effects on memory (Cohen’s *d* = 0.38), executive function (Cohen’s *d* = 0.40), and attention and processing speed (Cohen’s *d* = 0.33). No adverse effects were reported. Overall, yoga interventions for older adults were shown to be a safe, feasible, effective alternative practices for maintenance of cognitive function and prevention of cognitive decline particularly as effect sizes obtained were similar to those observed in RCTs testing the efficacy of cholinesterase inhibitors in individuals with similar level of cognitive impairment [17••].

More studies have evaluated the effects of Tai Chi on cognition. A recent 2020 meta-analysis analyzed the effects of Tai Chi on individuals aged 60 and old with mild cognitive impairment (MCI). This analysis included 11 RCTs showing Tai Chi could have moderate to significant benefits for global cognitive function (SMD = 0.35) in older adults with MCI, as suggested by previous studies [18••]. Regarding the specific domains of cognition, Tai Chi may improve to a small to medium or significant degree memory and learning (SMD = 0.37), mental speed and attention (SMD = 0.51), ideas, abstraction, figural creations, and mental flexibility (SMD = 0.29), and visuospatial perception (SMD = 0.29). More recently, a small feasibility study including 60 participants explored virtual reality-based Tai Chi interventions for older adults showed promising positive results of acceptance and promise for increased access and instruction with significant protective effect on abstract thinking and judgment, aerobic endurance, lower extremity endurance, balance, and gait speed but only the ability of abstract thinking and judgment was maintained for cognitive function in the VRTC group after 6 months [19]. Overall, Tai Chi produces low to moderate positive effects with low to minimal risk while being easily accessible and easy-to-learn.

There are many interventions that include components of mindfulness, but two programs with the largest evidence are mindfulness-based stress reduction (MBSR) and mindfulness-based cognitive therapy (MBCT). A recent RCT pilot study published in 2020 tested the safety and feasibility of MBSR in older adults with MCI and included 28 subjects. The study found that adults with MCI can safely participate and adhere to an MBSR program and described improved mindfulness skills, well-being, interpersonal skills, acceptance/awareness of MCI, and decreased stress reactivity [20]. Similarly, this supports the evidence found in several smaller RCT pilot studies showing positive results of mindfulness-based interventions on cognitive decline [21–23] Similarly, an RCT whose participants included older adults with stress disorders and subjective neurocognitive problems compared MBSR to health education, with the primary outcomes being memory and cognitive control [24]. Older adults receiving MBSR showed greater improvement in memory, but not cognitive control. Furthermore, the MBSR group improved on measures of worry, depression, and anxiety, and decreased cortisol level for those with high baseline cortisol. Although these studies demonstrate feasibility of MBSR with older adults with subjective cognitive decline (SCD) and MCI, and preliminary evidence for memory improvement, larger RCTs are needed to understand short and longer-term effects of MBSR and MBCT interventions and their influence on cognitive decline.

There is limited research on mindfulness interventions to specifically support caregivers, particularly those caring for individuals with neurocognitive disorders. Three RCTs have investigated the effects of mindfulness-based interventions in caregivers of persons with dementia [25–27]. One study divided 31 caregivers between an adapted MBCT (90-min sessions, 7 weeks) and two control groups: education and respite only [25]. Both MBCT and education enhanced the primary outcome of caregiver stress compared to the respite-only group. No effects were found on the secondary outcome measures of cognition and mindfulness. Another study randomized 78 caregivers of persons with dementia into a MBSR or active control group [26]. The MBSR group showed greater improvement in overall mental health, stress, and depression. Both interventions improved anxiety, social support, and burden. In another RCT, 38 family dementia caregivers were randomized to MBSR or standard social support control condition [27]. The caregivers in the MBSR group reported lower levels of perceived stress relative to active control group, but not at the 3-month follow-up. These findings for caregiver stress and well-being support prior evidence that MBSR and MBCT interventions can augment and benefit depression, anxiety, and stress-reduction [1].

## Aerobic Exercise

Aerobic physical activity is a promising therapy for the treatment and prevention of age-related cognitive decline and dementia. The American College of Sports Medicine recommends that exercise programs for older adults include both aerobic and nonaerobic physical activities, such as resistance training, balance training, and stretching, for optimal general health. A recent randomized controlled trial of 28 participants, including 14 with mild cognitive impairment (MCI) and 14 participants with Alzheimer’s disease (AD), were randomized to a control group and exercise group evaluated before and three months after the intervention on physical and cognitive abilities. The intervention included a 12-week multimodal physical exercise program on global cognition, executive function, and mobility in elderly people with MCI or AD. Results in this small study suggest that the 12-week multimodal physical exercise program contributed to improvements in the mobility and executive function of elderly individuals with MCI, but not of those with AD [28]. Currently, several larger scale RCTs are underway, but this suggests that physical exercise should be recommended to those in the early stages of dementia, including MCI, for more preventative measures.

Similarly, two recent meta-analyses examined the efficacy of exercise on cognition in older adults with MCI. In a 2018 meta-analysis, the authors looked at aerobic exercise (AE), resistance exercise (RE), and multimodal exercise and their effects on cognition. The results showed that physical exercise had beneficial effects for global cognition (SMD = 0.30, 95% confidence interval (CI): 0.10–0.49, *p* = 0.002). However, the effects of physical exercise on domain-specific cognitive function and psychological outcomes in MCI patients remain inconclusive [29]. In a more in-depth 2021 meta-analysis, the authors examined distinctive types of exercise, including AE and RE, since prior studies suggested distinctive types may exert different effects. A total of 71 trials with 5606 participants were included. All types of exercise were effective in increasing or maintaining global cognition, and resistance exercise had the highest probability of being the most effective intervention in slowing the decrease in global cognition (SMD = 1.05, 95% confidence interval (95%CI): 0.56–1.54), executive function (SMD = 0.85, 95%CI: 0.21–1.49), and memory function (SMD = 0.32, 95%CI: 0.01–0.63) in patients with cognitive dysfunction. The results support the beneficial effects of various exercise interventions on global cognition and revealed that RE had the highest probability of being the most promising exercise treatment to slow the decline of global cognition, executive function, and memory function for patients with cognitive dysfunction, especially for patients with dementia. For patients with MCI, multicomponent exercise (a combination of at least 2 types of exercise, such as AE, RE, and balance training) tended to be the most effective exercise therapy for preventing the decline of global cognition and executive function [30••].

MCI is a well-recognized risk factor for dementia and represents a critical window of opportunity for intervening and altering the trajectory of cognitive decline. Many studies have examined the effects of physical exercise interventions in MCI compared to moderate-severe dementia to better understand timing and impact of these interventions. A recent RCT including 494 individuals with mild-moderate dementia looked at the effects of moderate to high intensity exercise training on cognition. Unfortunately, a moderate to high intensity aerobic and strength exercise training program did not appear to slow cognitive impairment in people with mild to moderate dementia. However, the exercise training program improved physical fitness, but there were no noticeable improvements in other clinical outcomes [31]. In addition, there is an emerging evidence for the effect of low levels of physical activity or “sedentary behaviors” like sitting. A recent meta-analysis including 18 relevant cohort studies involving 250,063 participants and 2269 patients with dementia showed that sedentary behavior was significantly associated with increased risk of dementia (*RR* = 1.30; 95% CI: 1.12–1.51). The results suggest that sedentary behavior was independently associated with a significantly increased risk of dementia [32••]. Ultimately, more work is required to understand if physical activity recommendations for dementia prevention are the same as general “healthy aging” recommendations and when these interventions may be most beneficial. From the evidence presented, it appears that earlier interventions may be more beneficial with overall health benefits with exercise, regardless of effects on cognition.

## Nutrition and Dietary Interventions

Lifestyle interventions typically include diet and nutritional supplements. Dietary interventions address the effect of dietary choices on health and may include Western, traditional, and Mediterranean dietary patterns, as well as caloric restriction, among others. Both dietary and exercise interventions have strong evidence for its positive effects on cognition. The Mediterranean diet gained attention after a large, multicenter, randomized controlled trial, known as “the PREDIMED” study, showed that adherence to a Mediterranean diet can be a primary prevention strategy for cardiovascular disease and cognition [33, 34]. The Mediterranean diet entails the use of olive oil, high consumption of fruit, vegetables, legumes, cereals, and nuts. Additionally, it includes regular but moderate intake of wine (especially red wine) with meals, moderate consumption of fish, seafood, fermented dairy products (yogurt and cheese), poultry and eggs; as well as limited consumption of red and processed meats and sweets [1••]. Similar to the Mediterranean diet, the Dietary Approaches to Stop Hypertension (DASH) diet specifies a high consumption of plant-based foods but also limits the intake of saturated and total fat, cholesterol, and sodium; it was initially developed to target hypertension and cardiovascular risk factors. The Mediterranean-DASH Intervention for Neurodegenerative Delay (MIND) diet emphasizes natural plant-based foods as well as consumption of berries and green leafy vegetables while limiting animal foods and saturated fats. High adherence to all three diets may reduce Alzheimer’s Disease (AD) risk, with moderate adherence to the MIND diet also decreasing AD risk. Over the years, there has been strong, consistent evidence for the positive effects of these three dietary patterns on cognition and overall health and well-being in numerous randomized controlled trials and meta-analyses [1••].

## Vitamin Supplements

Antioxidant compounds such as vitamins A, C, and E play a significant role in the regulation of oxidative stress, a pathway associated with neurodegeneration and cognitive decline. However, vitamin E supplementation has not been found to have a protective effect against progression from mild cognitive impairment to dementia. Similarly, low folate levels and vitamin B status are linked to cognitive impairment during the aging process. Dementia workups often include measuring Vitamin B_12_ levels because deficiencies are considered as a reversible cause of cognitive decline. Various measures of vitamin B_12_, including methylmalonate, homocysteine, and cystathionine, are also associated with episodic memory deficits, slowed perceptual speed, and decreased total brain volume in adults aged 65 years and older [35]. Interestingly, data has related elevated homocysteine levels to an increased risk of cognitive impairment and dementia [2].

However, the evidence does not support a robust response in cognition with B vitamins. A multicenter RCT of adults over 50 years old with mild to moderate Alzheimer’s disease and normal folic acid, vitamin B_12_, and B_6_ levels showed no benefit or slowing of cognitive decline with folate, vitamin B_12_, and B_6_ supplementation [36]. Similarly, a Cochrane review found no short-term benefit from vitamin B_6_ supplementation in improving cognitive functions or mood [37]. The review found evidence that there is scope for increasing some biochemical indices of vitamin B_6_ status among older adults but potential effects on blood homocysteine levels were not assessed [37]. More randomized controlled trials are needed to study the possible benefits from vitamin B_6_ supplementation for older, healthy adults as well as those with cognitive impairment or dementia. Furthermore, the use of supplemental vitamins may come at a risk as shown in the Iowa Women’s Health Study, which demonstrated an increase in mortality with the use of various dietary supplements including vitamins B_6_ and B_9_ [38].

## Natural Products

Natural products typically include herbs, minerals, natural supplements, vitamins, and probiotics. A wide range of these products, with their neuroprotective properties, has been explored with regard to the treatment and prevention of neurocognitive impairment. In this review, we focus on the most commonly used natural remedies for neurocognitive disorders including omega-3 fatty acids, *S*-adenosyl methionine (SAMe), and nootropic gingko biloba.

### Omega-3 Fatty Acid Supplements

Omega-3 polyunsaturated fatty acids are a family of lipids that include eicosapentaenoic acid (EPA) and doxosahexaenoic acid (DHA), both derived from oily fish, as well as alpha-linolenic acid (ALA), derived from plant sources. Omega-3 fatty acids are essential for brain development and are a significant component of neuronal membranes in the brain. Maintaining sufficient levels of omega-3 fatty acids may support the integrity of the brain’s neurons and enhance synaptic neuroplasticity. Additionally, omega-3 fatty acids improve brain function through their antioxidant and anti-inflammatory effects, ultimately slowing down the progression of dementia.

The evidence regarding omega-3 fatty acids supplementation is difficult to ascertain due to the heterogeneity among studies, particularly regarding the omega-3 doses, preparations, and overall study design. Several meta-analyses examined their efficacy for the treatment of dementia. The most recent study was a Cochrane review that looked at the effects of omega-3 fatty acids to treat cognitive dysfunction. Six hundred thirty-two participants with mild to moderate Alzheimer’s disease were examined over 6, 12, and 18 months [39]. Their results showed that there was no evidence of an increased benefit from omega-3 fatty acids supplementation on cognitive function when measured at six months with the Mini-Mental State Examination (mean different (MD) 0.18, 95% CI − 1.05 to 1.41; 202 participants; 2 studies; high-quality evidence) or the Alzheimer’s Disease Assessment Scale-Cognitive subscale (SMD − 0.02, 95% confidence interval (DI) − 0.19 to 0.15; 566 participants; 3 studies; high-quality evidence) [39]. There was also no evidence of a benefit from omega-3 fatty acids on activities of daily living (SMD − 0.02, 95% CI − 0.19 to 0.16; 544 participants; 2 studies; high-quality evidence) [39]. Another Cochrane Database systematic review assessed the effects of omega-3 fatty acids supplementation on the prevention of dementia and cognitive decline in cognitively healthy older people [40]. Information was available from 3 RCTs, including 3536 participants in total [40]. This meta-analysis found no evidence to support a preventative effect following 24 or 40 months of intervention [40]. Longer-term studies may be needed to detect the possible effects and consequences of omega-3 fatty acid supplementation in preventing cognitive decline [40]. Other RCTs suggest that selected patients with an MMSE score > 27 were more likely to identify a positive effect of omega-3 fatty acids supplementation [41].

Typical doses range from 1 to 2 g per day with use of preparations ≥ 60% EPA in omega-3 fatty acids combinations [42••]. Potential side effects may include mild nausea and a fishy aftertaste. The previous concerns regarding the increased risk of bleeding associated with omega-3 fatty acids were disproved; however, caution is recommended in older adults taking anticoagulants [42••].

### S-Adenosyl-L-Methionine

*S*-adenosyl-l-methionine (SAMe) is a compound normally synthesized in the body and is formed from the amino acid l-methionine as part of a multi-step metabolic pathway involving folic acid and vitamin B_12_ [42••]. SAMe plays a role in depression as it is a necessary cofactor for the synthesis of serotonin, norepinephrine, and dopamine [42••]. There is evidence of SAMe deficiency in cerebrospinal fluid (CSF) in diseases such as depressive disorders, Alzheimer’s dementia, Parkinson’s disease, as well as rare inherited defects in folate and methionine metabolism [43]. However, there are very limited studies examining the contribution of SAMe on cognition independent of other nutraceuticals (SAMe and other vitamins). Conversely, there is more and significant evidence for the use of and benefit of SAMe for major depressive disorder (MDD), which is commonly associated with cognitive impairment. In preclinical and early clinical trials, there is some beneficial effects observed from SAMe alone, or in combination with other nutraceuticals, on cognitive function [43]. A secondary analysis from a RCT using SAMe for MDD (*n* = 46) showed that SAMe improved two memory-related cognitive functions (recall, *p* = 0.04 and word finding, *p* = 0/09), suggesting that SAMe may have beneficial effects on memory-related cognition in MDD [44].

### Ginkgo Biloba Supplements

Ginkgo biloba leaf extract is considered the most widely sold herbal dietary supplements in the USA [2]. Its professed biological effects include scavenging free radicals, reducing oxidative stress, decreasing neural damage, lowering platelet aggregation, as well as anti-inflammatory, antitumor, and antiaging activities [2]. Ginkgo biloba is typically classified as a therapeutic agent for dementia as it enhances cognitive function, including memory, learning ability, and abstract thinking [45]. No standards or guidelines regulate the constituent components of Ginkgo biloba leaf extract, nor are exposure limits imposed [2].

Over 30 placebo-controlled, double blind RCTs of Ginkgo biloba in patients with various types of dementia have yielded contradictory results [42••]. A meta-analysis by Hashiguchi et al. examined nine out of thirteen studies lasting between 12 and 52 weeks with doses > 120 mg per day in 2381 participants. In seven out of the nine studies, Ginkgo outperformed placebo. For Alzheimer’s dementia and vascular dementia subgroups, the advantage of Ginkgo was preserved over placebo. Another meta-analysis by Yang et al. found that Gingko biloba in combination with conventional medicine was superior in improving MMSE scores at 24 weeks for patients with Alzheimer’s disease and MCI as well as activities of daily living [46]. Doses of 240 mg per day appeared to be most effective with good tolerability. Ginko biloba has also been studied in treatment of behavioral and psychological symptoms of dementia and was noted to be superior over placebo in reducing neuropsychiatric symptoms except for delusions, hallucinations, and elation/euphoria [47••].

The suggested dose of Ginkgo is 120–240 mg/day, dosed two to three times per day [42••]. As with other natural products, Ginkgo biloba comes with risks. Caution is recommended when using *Ginkgo biloba* in those at increased risk of hemorrhage or taking anticoagulant medications as it may potentiate bleeding by inhibiting platelet-activating factor (PAF). Nevertheless, a recent meta-analysis of 18 trials including patients with dementia and healthy volunteers, diabetes mellitus, and peripheral artery disease did not find an added risk of bleeding based on hemostatic outcomes [48]. Side effects include mild GI upset, headache, irritability, dizziness, or allergic reactions [42••]. Thus, short-term use of Ginkgo biloba is acceptable under some conditions, dependent on clinician–patient discussions, with the potential risks being considered [42••].

## Other Complementary Approaches

Other complementary approaches include traditional Chinese medicine (TCM including acupuncture), Ayurvedic medicine, and body-based practices.

### Acupuncture

Acupuncture is a non-pharmacologic treatment that is based in traditional Chinese medicine. The theory of acupuncture is founded on scientifically, non-detectable, energy pathways known as meridians that are interconnected within the body whereby thousands of acupoints along these pathways can be stimulated—most often by inserting thin needles through the skin [49]. Acupuncture’s mechanism of action is not fully understood but it is thought to stimulate the central nervous system through the release of specific neurotransmitters and hormones [49].

Acupuncture has been studied in Alzheimer’s disease and vascular dementia and was found to be safe and reliable for improving cognitive function. Additionally, the use of acupuncture for treatment of behavioral and psychological symptoms of dementia is effective, tolerable, and safe. A systematic review by Harris et al. showed that acupuncture therapy led to statistically significant improvements in activities of daily living, agitation, anxiety, depression, neuropsychological disturbances, and sleep disturbances [49]. However, it was noted that variation in study designs and outcomes often limited the interpretation about acupuncture’s overall effectiveness[49]. Furthermore, numerous reports of positive effects of acupuncture for cognitive and psychiatric symptoms of vascular dementia have emerged from China, but a 2007 Cochrane review found the methodologies of most of these studies to be highly problematic, leaving no convincing evidence of acupuncture’s positive benefit and the effectiveness [50]. More clinical studies investigating the use acupuncture in dementia are needed to further establish its efficacy.

### Ayurvedic Medicine

Ayurveda, which translates as “the science of life,” is a complete natural health care system that began in India more than 5000 years ago. It incorporates an individualized regimen for patients that includes diet, meditation, herbal preparations, and other techniques. However, there are no extensive controlled trials of Ayurveda in older adults. A major limitation with the use of Ayurvedic herbal products is hepatotoxicity and heavy metal poisoning that has been continuously reported with their use [51]. Around 20–22% of US and Indian manufactured Ayurvedic medicines that were retrieved from the Internet in 2005 contained detectable lead, mercury, or arsenic [51, 52]. Despite these reports, this form of treatment is widely utilized by the majority in rural India [51].

### Body-Based Practices

Body-based practices may include spinal manipulation (e.g., chiropractic, osteopathic medicine), aromatherapy, and massage therapy. Evidence for these interventions in neurocognitive disorders have been explored but remain more limited. Aromatherapy, a therapy related to massage, relies on the use of essential oils extracted from plants for health purposes. At times, aromatherapy combines the scent of essential oils with calming effects (e.g., lavender). Aromatherapy has been examined for its effects on agitation in patients with dementia and demonstrated significant reduction of agitated behaviors and depressive mood overall. Additionally, one study showed that the combination of aromatherapy and massage tended to alleviate agitated behaviors and depressive symptoms in dementia as compared to either cognitive stimulation therapy or reminiscence therapy alone [53]. One randomized controlled trial studied the effectiveness of lavender and lemon balm (*Melissa officinalis*) essential oils on agitated behaviors of older adults with and without dementia residing in a residential facility [54••]. They found that lemon balm was more effective in reducing agitated behaviors in residence without dementia while lavender was more effective in reducing agitated behaviors in those with dementia [54••]. Another double-blind, placebo-controlled trial used lemon balm oil (*Melissa officinalis*) for managing agitation in severe dementia and found overall improvement in agitation [55]. Although some risks of respiratory pathways’ irritation exist in individuals with underlying lung conditions, aromatherapy can be a safe and effective intervention to alleviate clinically significant agitation in older adults with severe dementia.

## Conclusion

Although there is increasing interest and use of CIM, particularly in older adults, there is still insufficient data on the prevalence, effectiveness, efficacy, safety, and health economic benefits of most CIM treatments for this age group. There is strongest and most consistent evidence about the benefits of lifestyle interventions and mind–body therapies for cognitive disorders, with less evidence for caregiver stress and well-being. However, the non-invasive nature of mind–body therapies and lifestyle modifications make these interventions make them more favorable, especially when compared to the limited evidence and higher potential for drug-drug interactions with supplementation and herbal use. Another significant advantage of including lifestyle interventions is the active participation of the individuals and their family caregivers in managing their own health and well-being, which can also increase adherence to the health practices. Gaining a better understanding of the clinical effects of CIMs, as well as their safety, is critical for the management of neurocognitive disorders. Overall, CIM therapies for older adults is a promising area of research, particularly as these interventions can be seen as a more preventative and oriented toward well-being and healthy aging.
